# Structure of Heteropentameric GABA_A_ Receptors and Receptor-Anchoring Properties of Gephyrin

**DOI:** 10.3389/fnmol.2019.00191

**Published:** 2019-08-07

**Authors:** Vikram Babu Kasaragod, Hermann Schindelin

**Affiliations:** Institute of Structural Biology, Rudolf Virchow Center for Experimental Biomedicine, University of Würzburg, Würzburg, Germany

**Keywords:** GABA_A_ receptors, gephyrin, diazepam, GABA, PIP_2_, artemisinin, Cryo-EM, inhibitory neurotransmission

## Abstract

γ-Aminobutyric acid type A receptors (GABA_A_Rs) mediate the majority of fast synaptic inhibition in the central nervous system (CNS). GABA_A_Rs belong to the Cys-loop superfamily of pentameric ligand-gated ion channels (pLGIC) and are assembled from 19 different subunits. As dysfunctional GABAergic neurotransmission manifests itself in neurodevelopmental disorders including epilepsy and anxiety, GABA_A_Rs are key drug targets. The majority of synaptic GABA_A_Rs are anchored at the inhibitory postsynaptic membrane by the principal scaffolding protein gephyrin, which acts as the central organizer in maintaining the architecture of the inhibitory postsynaptic density (iPSD). This interaction is mediated by the long intracellular loop located in between transmembrane helices 3 and 4 (M3–M4 loop) of the receptors and a universal receptor-binding pocket residing in the C-terminal domain of gephyrin. In 2014, the crystal structure of the β3-homopentameric GABA_A_R provided crucial information regarding the architecture of the receptor; however, an understanding of the structure and assembly of heteropentameric receptors at the atomic level was lacking. This review article will highlight recent advances in understanding the structure of heteropentameric synaptic GABA_A_Rs and how these structures have provided fundamental insights into the assembly of these multi-subunit receptors as well as their modulation by diverse ligands including the physiological agonist GABA. We will further discuss the role of gephyrin in the anchoring of synaptic GABA_A_Rs and glycine receptors (GlyRs), which are crucial for maintaining the architecture of the iPSD. Finally, we will also summarize how anti-malarial artemisinin drugs modulate gephyrin-mediated inhibitory neurotransmission.

## Introduction

Complex macromolecular interplays at excitatory and inhibitory synapses contribute in a fundamental way to the incredible functional capabilities of the human brain. Inhibition in the central nervous system (CNS) is mediated by key members of the Cys-loop receptor superfamily, in particular, the γ-aminobutyric acid type A receptors (GABA_A_Rs), and, to a smaller extent, the glycine receptors (GlyRs). Synaptic GABA_A_Rs are pentameric ligand-gated ion channels (pLGICs) mainly composed of two α, two β and a single γ subunit, which are selected from a diverse pool of 19 different subunit types (Sigel and Steinmann, 2012). Each subunit consists of an extracellular domain (ECD) rich in β-sheet architecture, a four α-helical bundle transmembrane domain (TMD) and two intracellular, unstructured loops, the short M1–2 and the long M3–4 loop, connecting these helices. The ECDs harbor the sites for the natural agonist GABA and drugs, in particular the benzodiazepines, while the binding site for allosteric modulators such as endogenous neurosteroids reside in the TMD (Miller and Aricescu, 2014; Laverty et al., 2017; Miller et al., 2017; Phulera et al., 2018; Zhu et al., 2018).

The majority of synaptic GABA_A_Rs, as well as GlyRs, are recruited to and anchored at the inhibitory postsynaptic membrane by the principal scaffolding protein gephyrin (Kirsch et al., 1991; Kneussel et al., 1999). This multi-domain protein consists of two terminal domains; the N-terminal G domain (GephG) and the C-terminal E domain (GephE), which are connected by a highly unstructured linker region (Kirsch et al., 1991; Prior et al., 1992; Schwarz et al., 2001; Kim et al., 2006; Sander et al., 2013). The interaction of gephyrin with postsynaptic receptors is mediated by a continuous segment within the large intracellular M3–4 loop and a universal receptor-binding pocket residing in GephE. In addition to the interactions with inhibitory neurotransmitter receptors, gephyrin also interacts with a diverse set of macromolecules, thus playing an essential role in establishing and maintaining the architecture of the inhibitory postsynaptic density (iPSD; Tyagarajan and Fritschy, 2014; Kasaragod and Schindelin, 2018). Besides its anchoring function, gephyrin also catalyzes the two terminal steps in the evolutionarily conserved molybdenum cofactor (Moco) biosynthesis pathway (Kuper et al., 2004; Kasaragod and Schindelin, 2016), a critical active site component of almost all Mo-containing enzymes.

Small molecules such as benzodiazepines, which target synaptic α-subunit containing GABA_A_Rs, have been in clinical use for decades for the treatment of neurological disorders (for a detailed review see Rudolph and Knoflach, 2011). Since dysfunctional inhibitory neurotransmission triggered by defects residing in either the receptors or gephyrin has been implicated in a diverse set of neurodevelopmental disorders including anxiety and epilepsy (Agarwal et al., 2008; Hales et al., 2013; Dejanovic et al., 2014, 2015), these macromolecules may be suitable targets of future structure-based drug discovery processes.

In this review article, we will highlight recent advances in the structural elucidation of heteromeric GABA_A_Rs and how these structures have helped us to understand the assembly and also regulation of these ion channels by diverse ligands (Laverty et al., 2019; Masiulis et al., 2019). Besides, we will also briefly discuss the alternative GABA_A_R/GlyR recruitment to the iPSD and finally, summarize our recent contribution on the elucidation of the modulation of inhibitory neurotransmission by artemisinins.

## Structural Insights Into Synaptic Heteropentameric GABA_A_Rs

Until recently, knowledge regarding the atomic architectures of GABA_A_Rs and their modulation by ligands was derived solely from structural studies performed with either homopentameric receptors or homopentameric receptor chimeras. While the crystal structure of the β3 homopentameric GABA_A_R described the architecture of the receptor for the first time (Miller and Aricescu, 2014), studies with chimeric versions of the GABA_A_Rs receptors provided atomic insights into the neurosteroid (e.g., pregnanolone and pregnenolone) binding site in the TMD and the modulation of GABA_A_Rs by these compounds (Laverty et al., 2017; Miller et al., 2017). Nevertheless, structures of heteropentameric receptors had remained elusive until recently, when several independent studies (Phulera et al., 2018; Zhu et al., 2018; Laverty et al., 2019; Masiulis et al., 2019), which were aided by recent developments in the field of cryo-electron microscopy (Cryo-EM), provided crucial insights into the structure of heteropentameric receptors.

**Figure 1:**
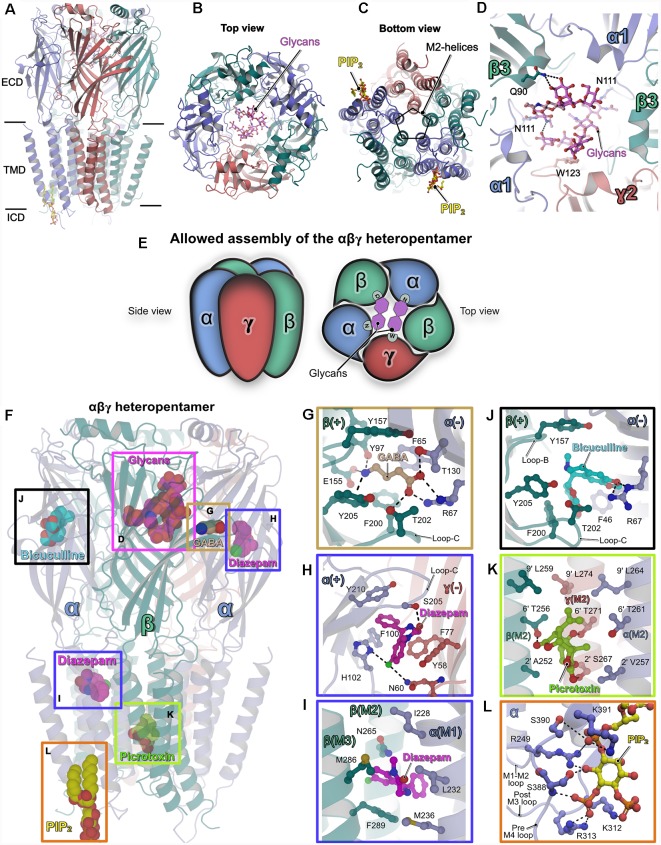
Structures of heteropentameric γ-aminobutyric acid type A receptors (GABA_A_Rs). **(A)** Side view of the overall structure of the heteropentameric GABA_A_R as determined by cryo-electron microscopy (Cryo-EM). **(B)** Architecture of the receptor viewed from the extracellular side (top view) with the receptor subunits in cartoon representation and the glycans in ball and stick representation. **(C)** View of the receptor from the intracellular side into the ion-conducting pore (bottom view). **(D)** Close-up view of the glycosylation sites in the extracellular vestibule. The glycans and critical residues mediating their binding are shown in ball and stick representation. **(E)** Schematic representation of the underlying principle governing the assembly of synaptic heteropentameric GABA_A_Rs. The scheme demonstrates how glycosylation of the conserved Asn111 plays a crucial structural role in receptor assembly, which in turn also determines the order in which the subunits are arranged. **(F–L)** Structures of GABA_A_Rs bound to various ligands. **(F)** The heteropentameric GABA_A_R is shown in cartoon representation along with structurally validated ligands in space-filling representation. Enlarged views of the binding pockets of the natural agonist GABA (PDB: 6HUJ, **G**), the positive allosteric modulator (PAM) diazepam (PDB: 6HUP, **H–I**), the competitive antagonist bicuculline (PDB: 6HUK, **J**), the channel blocker picrotoxin (PDB: 6HUG, **K**) and the lipid PIP_2_ (PDB: 6I53, **L**). Enlarged views are shown according to the color of the box in the overall structure displayed in **(F)**. In **(F–L)** all ligands and the critical residues which mediate binding are shown in ball and stick and the protein chains in cartoon representation.

The first Cryo-EM structure of a heteromeric GABA_A_R, in this case, composed of the human α1β2γ2 subunits, was determined by Hibbs and colleagues (Zhu et al., 2018). Subsequently, Gouaux and coworkers (Phulera et al., 2018) solved the Cryo-EM structure of the rat α1β1γ2 heteropentamer. Although both structures provided valuable insights into the binding of the agonist GABA and also the modulation of these receptors by flumazenil, which targets the benzodiazepine binding site, these structures were somewhat incomplete with respect to the overall architecture of the receptors. The first study (Zhu et al., 2018) described a structure in which the pore had collapsed due to an unusual arrangement of the γ2-subunit (PDB: 6D6U) while the other structure (Phulera et al., 2018) featured fragmented density in the TMD (PDB: 6DW0). A common denominator of these structures is that they were solved in the presence of detergents. Whereas Phulera et al. (2018) determined the structure by using the shorter splice variant of the γ2 subunit, it is unclear which γ2 subunit splice variant was used by Zhu et al. (2018). In addition, for the structural studies, Zhu et al. (2018) replaced the intracellular loop connecting the M3–4 helices with a seven-residue artificial linker, whereas Phulera et al. (2018) introduced a fluorescent tag in the M3–4 loop of the γ2 subunit in addition to shortening the M3–4 loops of the other subunits. In this review article, we will mainly focus on the structures of the human α1β3γ2 receptor published recently (Laverty et al., 2019; Masiulis et al., 2019) in which full-length GABA_A_R subunits were used and the structures were solved by reconstituting the receptors in discoidal membranes (nanodiscs) composed of a double layer of lipid molecules surrounded by a membrane scaffold protein. These structures yielded unprecedented insights not only into the overall architecture of heteropentameric GABA_A_Rs but also into the binding of diverse ligands including the agonist GABA. Finally, these structures also demonstrated how membrane lipids interact with the TMD (Figures 1A–D).

All structures revealed that the subunits are arranged in an α–β–α–β–γ arrangement in a clockwise manner when viewed from the extracellular side, consistent with previous biochemical studies (Tretter et al., 1997; Baumann et al., 2002). Although the earlier structural analyses (Phulera et al., 2018; Zhu et al., 2018) and the more recent ones (Laverty et al., 2019; Masiulis et al., 2019) differed in receptor subunit composition and structural organization of the TMD, a common denominator amongst all of them was the observation of two unique glycosylation sites in the extracellular vestibule. These glycosylations originate from residue Asn111 which is present in all α-subunits and hence all heteropentameric GABA_A_Rs. In addition to several inter-glycan interactions, Gln90 in the β-subunit mediates interactions with these glycans *via* hydrogen bonds which are augmented by a critical hydrophobic π-π stacking interaction with the conserved residue Trp123 residing in the γ2 subunit (Figure 1D). Depending on their occupancies, these glycans may have critical implications on the assembly and subunit arrangement in heteropentameric GABA_A_Rs. Interestingly, a recent study (Hannan and Smart, 2018) showed that α1 homopentamer formation is controlled by two TMD residues (Gln241 and Ala290); if either residue is mutated (Q241W or A290W), α1 forms functional homopentamers on the surface of HEK cells. In addition, future research will also be required to understand the mechanism of assembly of heterodimeric receptors and the impact of glycosylation of Asn111 on receptor assembly. Nevertheless, this post-translational modification (PTM) is unique to heteropentameric GABA_A_Rs and may have critical implications for receptor permeability while also critically contributing to subunit composition and arrangement within the heteropentamer (Figure 1E). In addition to this crucial information regarding the assembly of the heteropentamers, a series of structures of the α1β3γ2-GABA_A_R in complex with diverse ligands provided valuable insights into their interactions with these receptors as briefly described below (Figures 1F–L).

### GABA

The agonist GABA only occupied the two orthosteric binding sites created by the contribution of the principal β-subunit and complementary α-subunit as already reported in one of the earlier structures (Zhu et al., 2018), however, this is in contrast to the three GABA binding sites proposed by the Gouaux group (Phulera et al., 2018). The binding of GABA is mediated by residues from the “aromatic box” created by Tyr157, Phe200, Tyr205 from the β3-subunit and Phe65 from the α1-subunit, which are located in the ECD at the β–α subunit interface. The agonist is stabilized by an extensive hydrogen-bonding network between GABA and Tyr97, Glu155 of the principal β-subunit along with Arg67 and Thr130 from the complimentary α-subunit. The contribution from loop-C, through Thr202 *via* a hydrogen bond with the GABA carboxylate, additionally stabilizes the agonist (PDB: 6HUJ; Figure 1G).

### Diazepam

Diazepam, which acts as a positive allosteric modulator (PAMs), has been used clinically for decades in the treatment of anxiety disorders and also epilepsy (Rudolph and Knoflach, 2011). The structure of the GABA_A_R-diazepam complex (PDB: 6HUP) revealed that the drug molecule not only binds to the “classical diazepam binding pocket” created by the principal α-subunit and the complementary γ-subunit, but, in addition, a strong density feature was observed in the TMD. The binding at the ECD (Figure 1H) is mediated mainly by hydrophobic π-π stacking interactions with Phe100, His102 from the principal α-subunit and Phe77 and Tyr58 from the complementary γ-subunit. In addition, hydrogen bonds from His102 (α-subunit) and Asn60 (γ-subunit) augment diazepam binding at the ECD. Strikingly, His102 has been shown to be critical for the binding of benzodiazepine. Heteropentameric receptors composed of the αβγ subunits and containing either the α1-α3 or α5 subunits possess this histidine and are benzodiazepine-sensitive. In contrast, in the α4 and α6-subunits an arginine is present at this position and the corresponding receptors are non-responsive to benzodiazepine (Wieland et al., 1992; Davies et al., 1998; Dunn et al., 1999).

In contrast, the binding of diazepam in the TMD is mediated by the M2 and M3 helices from the β-subunit as well as the M1 helix from the α-subunit. Previous studies have proposed this site as target area of anesthetics such as azietomidate (Forman and Miller, 2011). The binding is mediated purely by hydrophobic interactions involving Met286 and Phe289 from M3 of the β-subunit as well as Leu232 and also Met236 from M1 of the α-subunit. In addition, the drug molecule comes into close proximity of Asn265 from the M2 helix of the β-subunit, which, in turn, will have a direct impact on the gating properties of the GABA_A_R pore (Figure 1I). The two diazepam binding sites may provide an explanation for the biphasic potentiation of these receptors by diazepams as observed in electrophysiological experiments (Walters et al., 2000). Nevertheless, future research will be required to fully understand the properties of the secondary diazepam-binding site located in the TMD.

### Bicuculline

The action of the competitive antagonist bicuculline is achieved by its binding into the aromatic box with contributions from loop-B and loop-C of the principal β-subunit (PDB: 6HUK). Bicuculline is sandwiched between the aromatic Tyr157 from loop-B of the principal β-subunit and Phe46 from the complementary α-subunit. In addition, hydrogen bonds to the guanidinium group of Arg67, which is also critical for agonist-binding, mediate binding of this antagonist (Figure 1J).

### Picrotoxin

The structural analyses also revealed the binding site and blocking mechanism of GABA_A_Rs by the classical channel blocker picrotoxin (Figure 1K). The picrotoxin-binding pocket resides in the channel and is lined by the Leu at the 9′ position (Leu264, Leu259 and Leu274 from the α, β and γ-subunit, respectively) and the respective variable 2′ residues (Val257, Ala252 and Ser267 from the α, β and γ-subunits, respectively) of the M2 helices in each subunit. In addition, hydrogen bonds mediated by the 6′ residues (Thr261 Thr256 and Thr271 from the α, β and γ-subunits, respectively), with principal contributions from the β and γ subunits, strengthen picrotoxin-binding (PDB: 6HUG).This is in contrast to the glutamate-gated chloride channel (GluCl), in which the picrotoxin-induced channel block is achieved by its binding into a pocket created by the 2′-Thr and -2′-Pro residues (Hibbs and Gouaux, 2011).

### Phosphatidylinositol Phosphates

The GABA_A_R structure embedded in a lipid bilayer also revealed binding sites for phosphatidylinositol 4,5 bisphosphate (PDB: 6I53). The lipid occupies an electropositive area exclusive to the α-subunits and its binding is mediated by extensive hydrogen bonds from Lys312 and Arg313 from the post-M3 loop as well as Ser388, Ser390 and Lys391 from the pre-M4 loop with the inositol head group. PIP_2_ binding is also complemented by Arg249 from the M1–2 loop (Figure 1L). Interestingly, while Lys312 and Arg313 are conserved in all synaptic α-subunits, the remaining residues mediating PIP_2_-binding are conserved only in synaptic α-subunits (α1–3 and α5) and not in extrasynaptic α-subunits (α4 and α6). Thus, this specificity of synaptic GABA_A_Rs towards PIP_2_ may have critical implications for receptor trafficking at the iPSDs and on the channel gating properties as seen in the structurally validated cases of the transient receptor potential vanilloid 5 (TRPV5; Hughes et al., 2018), TRP mucolipin 1 (TRPML1; Fine et al., 2018) and also inward rectifier potassium channels (Hansen et al., 2011).

## Artemisinins—Gephyrin-Specific Modulators of Inhibitory Neurotransmission

**Figure 2:**
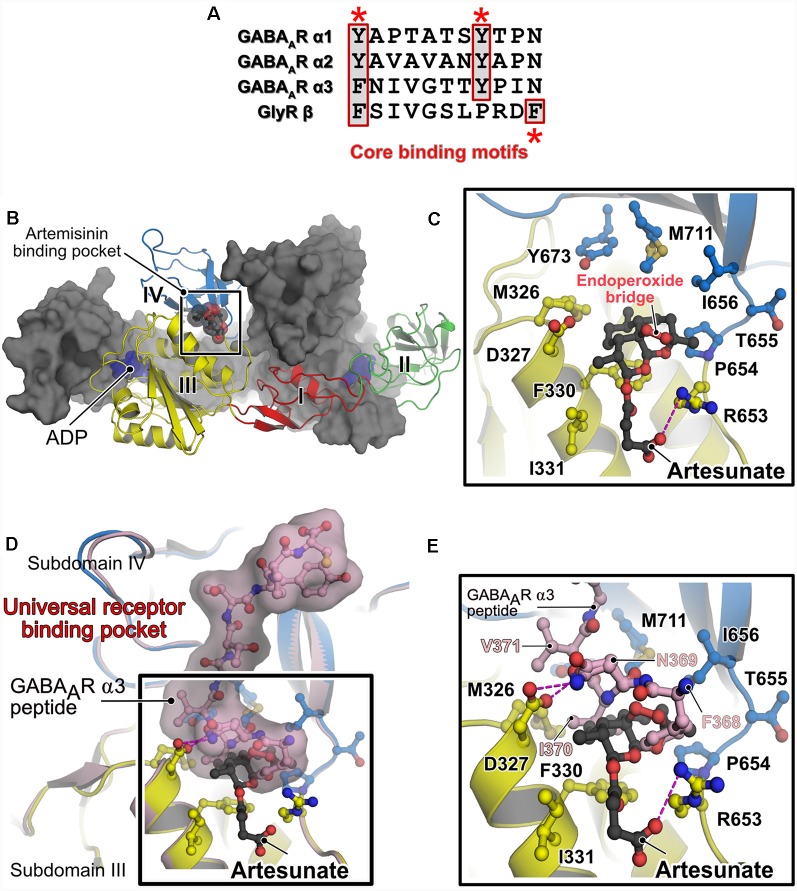
Alternative receptor clustering of the GABA_A_Rs by gephyrin and modulation by artemisinins. **(A)** Sequence alignment of the core binding motifs located in the M3–M4 loops of the glycine receptor (GlyR) β and GABA_A_Rs α1, α2 and α3 subunits. Structurally conserved aromatic residues are highlighted with red asterisks. The multiple sequence alignment is represented by using the ESPript server (Robert and Gouet, 2014). **(B)** Crystal structure of GephE in complex with the anti-malarial drug artesunate (PDB: 6FGC). One protomer of the dimeric E domain is shown in cartoon representation, with the four subdomains (indicated by Roman numerals) being colored differently. The second protomer is shown in surface representation in gray. The bound artesunate is shown in space-filling representation. **(C)** Enlarged view of the artesunate-binding pocket demonstrating that binding is mediated by residues present in subdomains III and IV of GephE. The bound artesunate and residues which mediate binding are shown in ball and stick representation. **(D)** Superimposition of the crystal structures of GephE in complex with artesunate (PDB: 6FGC) and the GephE-GABA_A_R-α3 subunit-derived peptide complex (PDB:4U90). **(E)** An enlarged view of the binding pocket of artemisinin or the N-terminal end of the peptide demonstrates that artesunate inhibits critical contacts (^368^FNI^370^) between the receptor and GephE.

The central scaffolding protein gephyrin anchors a large subset of postsynaptic GABA_A_Rs (mainly those containing the α1-3 subunits) and also heteropentameric GlyRs, *via* their β-subunit, to the iPSD. This interaction is mediated by the universal receptor-binding pocket residing in the C-terminally located GephE domain and the M3–4 loop of the cognate inhibitory receptor (Maric et al., 2011). Common determinants between GABA_A_Rs and the GlyR are the presence of an aromatic Phe/Tyr at the first position of the core binding pocket and a conserved Tyr at position 8 in the cognate GABA_A_R subunits (Kim et al., 2006; Tretter et al., 2008, 2011; Maric et al., 2011, 2014a,b, 2015; Mukherjee et al., 2011; Figure 2A). Both types of receptors bind to a hydrophobic groove in GephE generated by contributions from subdomains III and IV. Although these receptors bind to an overlapping binding pocket and engage in similar interactions at the N-terminus of the core-binding motif, a receptor-specific interaction is present at the C-terminus. As could be only derived from the crystal structures (GephE-GlyRβ-49, Kim et al., 2006 and GephE-GABA_A_R α3, Maric et al., 2014a), the Tyr at the +8 position of GABA_A_R α3 subunits correspond to a Phe located at the last position of the GlyR β-subunit.

Recently, the anti-malarial drug artemisinin and its semi-synthetic derivatives, collectively referred to as artemisinins, were discovered to target GABA_A_R signaling by interacting with gephyrin in pancreatic cells. While one study concluded that this interaction mediates the trans-differentiation of glucagon-producing Tα cells into insulin-secreting Tβ cells, thus ascribing an anti-diabetic nature to these compounds (Li et al., 2017), subsequent studies (van der Meulen et al., 2018; Ackermann et al., 2018) failed to reproduce the induction of trans-differentiation in pancreas-derived cells. Chemically, artemisinins are sesquiterpene lactones with an unusual endoperoxide bridge. In traditional Chinese medicine, artemisinins have been used for centuries to treat malaria and artemisinin-based combination therapies (ACTs) such as artesunate, the succinate derivative of artemisinin, with lumefantrine and artemether together with mefloquine are recommended by the World Health Organization (WHO, 2015) as standard drug regiment to treat malaria caused by *Plasmodium falciparum*. In addition to their anti-parasitic activity, artemisinins have additionally been implicated in regulating the activity of multiple cellular pathways, including the modulation of a variety of cancers (Crespo-Ortiz and Wei, 2012; Tu, 2016). Despite the widespread applications of these compounds as drugs and effectors of cellular pathways, the molecular basis of their regulatory properties including their target recognition mechanisms has so far remained elusive.

Studies from our lab deciphered the molecular basis for the interaction between gephyrin and artemisinins by determining the first structure of a protein-artemisinin complex (Kasaragod et al., 2019; Figure 2B). Specifically, we determined crystal structures of GephE with the artemisinin derivatives artesunate and artemether. The structures revealed that artemisinin-binding is mediated by a hydrophobic pocket formed by contributions from subdomains III and IV of GephE (Figure 2C). More importantly, these structures revealed that these compounds target the N-terminal region of the universal receptor-binding pocket in GephE and inhibit important hydrophobic interactions (^368^FNI^370^ of the GABA_A_R α3 subunit and ^398^FSI^400^ of the GlyR β subunit), which represent critical determinants of the gephyrin-receptor interactions containing the aromatic residues at the first position of the consensus binding motif (Figures 2D,E). Displacement isothermal titration calorimetry (ITC) measurements and a supported membrane sheet assay (SCMS) demonstrated that these compounds negatively affect the gephyrin-receptor interaction. Electrophysiological experiments revealed a significant decrease in glycinergic currents in the presence of these compounds, with a strict dependence on gephyrin. Furthermore, receptor and gephyrin clustering studies displayed a strong and time-dependent decrease in GABA_A_R and gephyrin cluster sizes. In addition, our analyses also revealed a time-dependent neurotoxic effect of these compounds, in line with previous observations of cytotoxic effects of these compounds when administered in high doses (Brewer et al., 1994; Wesche et al., 1994). Since artemisinins have been shown to be capable of crossing the blood brain barrier (Davis et al., 2003) and as dysfunctions in gephyrin-mediated neurotransmission have been implicated in severe neurological disorders such as Alzheimer’s disease, autism, schizophrenia, epilepsy and also in hyperekplexia (Agarwal et al., 2008; Fang et al., 2011; Hales et al., 2013; Dejanovic et al., 2014, 2015), the gephyrin-artemisinin co-crystal structures may serve as a starting point for future drug development efforts against these disorders. In addition, the discovery of the artemisinin-binding pocket may serve as the basis for the future identification of additional cellular artemisinin-targets *via in silico* approaches. This study also established artemisinins as a tool for impairing inhibitory neurotransmission, which could eventually help to better understand the physiology of the human brain.

## Conclusions and Future Perspectives

Despite a plethora of high-resolution structures of GABA_A_Rs these receptors, initially homopentameric, but recently, driven by Cryo-EM, also heteropentameric receptors, a complete understanding of the multiple architecture and function of the iPSD still remains elusive. First and foremost, will be to address the lack of structures of extrasynaptic GABA_A_Rs. The structural elucidation of such a variant will certainly reveal whether these receptors also follow the same assembly principle as that observed for synaptic GABA_A_Rs. Furthermore, all currently available structural information on inhibitory neurotransmitter receptors was determined for receptors in the absence of any binding partners. In the context of the iPSD, one should take into consideration that these receptors are closely associated with scaffolding proteins such as gephyrin (Kneussel et al., 1999) and collybistin (Kins et al., 2000; Saiepour et al., 2010) as well as with the auxiliary subunit GARLH (Davenport et al., 2017; Yamasaki et al., 2017). While most receptor structures were determined by shortening the unstructured M3–M4 loop (Miller and Aricescu, 2014; Phulera et al., 2018; Zhu et al., 2018), the most recent studies were performed with full-length heteropentameric GABA_A_Rs including the native M3–M4 loop (Laverty et al., 2019; Masiulis et al., 2019). Nevertheless, even in these latest structures, these residues could not be resolved. At the iPSD, this region serves as the interaction hub for intracellular binding partners and hence the full-length heteropentameric receptors provide the necessary framework for structural studies with intracellular binding partners such as gephyrin and collybistin. The elucidation of the macromolecular complexes involving the receptors and their intracellular binding partners will provide crucial information not only regarding the structural organization of the intracellular loops but will also generate a molecular understanding of receptor clustering by scaffolding proteins at the iPSD. Hence, future research should be directed towards achieving a holistic, high-resolution view of the iPSD.

Another critical aspect is that, although the structure of the GephE-GABA_A_R α3-derived peptide complex provides critical information about the alternative receptor recruitment by gephyrin, high-resolution structural data describing how different types of GABA_A_Rs are recruited and anchored at the iPSD is still missing. The membrane sheet assay employed to study the inhibitory effect of artemisinins can also be adopted to analyze these uncharacterized GABA_A_Rs as it will take into consideration possible avidity effects triggered by the presence of two gephyrin-binding α-subunits in the heterotrimeric GABA_A_Rs and the oligomeric state of gephyrin as well as membrane contributions to the gephyrin-receptor interaction. With respect to the function of gephyrin, crucial information regarding the mechanism of the oligomeric organization of this scaffolding protein is still missing.

Although our structures of GephE-artemisinin complexes provide valuable insights into the modulation of inhibitory neurotransmission by gephyrin, multiple aspects of the regulation still remain to be deciphered; (a) What are possible effects of artemisinins on presynaptic terminals? (b) How does the balance of inhibitory and excitatory neurotransmission counteract the administration of artemisinins in human patients? (c) Are artemisinin metabolites equally potent as their parental compounds in modulating inhibitory neurotransmission? Although our structures can be used for the development of gephyrin-specific regulators of neurotransmission, one has to bear in mind that artemisinins influence a variety of cellular pathways possibly targeting multiple proteins. Thus, future structure-based drug design studies to optimize this lead compound with the aim of increasing its specificity towards gephyrin should be conducted. At the same time, structures of these compounds with other cellular targets would be desirable to better understand the molecular mechanism underlying target recognition and the pharmacological action of these anti-malarials.

## Author Contributions

VK prepared the figures and illustrations. VK and HS wrote the manuscript.

## Conflict of Interest Statement

The authors declare that the research was conducted in the absence of any commercial or financial relationships that could be construed as a potential conflict of interest.
